# Consistent and chronic cochlear implant use partially reverses cortical effects of single sided deafness in children

**DOI:** 10.1038/s41598-020-78371-6

**Published:** 2020-12-09

**Authors:** Hyo-Jeong Lee, Daniel Smieja, Melissa Jane Polonenko, Sharon Lynn Cushing, Blake Croll Papsin, Karen Ann Gordon

**Affiliations:** 1 Archie’s Cochlear Implant Laboratory, Hospital for Sick Children, Rm 6D08, 555 University Ave, Toronto, ON M5G 1X8 Canada; 2 Department of Communication Disorders, Hospital for Sick Children, Toronto, ON Canada; 3 Institute of Medical Science, University of Toronto, Toronto, ON Canada; 4 Department of Otolaryngology-Head and Neck Surgery, Hospital for Sick Children, Toronto, ON Canada; 5 Department of Otolaryngology-Head and Neck Surgery, University of Toronto, Toronto, ON Canada; 6 Department of Otorhinolaryngology-Head and Neck Surgery, Hallym University College of Medicine, Chuncheon, Republic of Korea

**Keywords:** Cortex, Paediatric research, Disability

## Abstract

Potentially neuroprotective effects of CI use were studied in 22 children with single sided deafness (SSD). Auditory-evoked EEG confirmed strengthened representation of the intact ear in the ipsilateral auditory cortex at initial CI activation in children with early-onset SSD (n = 15) and late-onset SSD occurring suddenly in later childhood/adolescence (n = 7). In early-onset SSD, representation of the hearing ear decreased with chronic CI experience and expected lateralization to the contralateral auditory cortex from the CI increased with longer daily CI use. In late-onset SSD, abnormally high activity from the intact ear in the ipsilateral cortex reduced, but responses from the deaf ear weakened despite CI use. Results suggest that: (1) cortical reorganization driven by unilateral hearing can occur throughout childhood; (2) chronic and consistent CI use can partially reverse these effects; and (3) CI use may not protect children with late-onset SSD from ongoing deterioration of pathways from the deaf ear.

## Introduction

The aims of the present study were to: (1) examine cortical effects of single sided deafness (SSD) occurring either early or acquired later in childhood and (2) to determine whether these effects can be reversed by provision of a cochlear implant (CI) in the deaf ear.

Children with SSD do not have access to binaural (two-eared) hearing which is the foundation for localizing sounds in space. With impaired sound localization^1,2^, they are not able to use spatial separation to distinguish between multiple sound sources, compromising their ability to listen in noise^2,3^. Developmental effects of SSD in children include impairments in spoken language^4–6^ and slower rates of educational progress^7–9^. In a recent study, we found that children with early-onset SSD have impaired visuo-spatial memory which impacts their reading, mathematics, and receptive language^10^. Even a mild degree of asymmetric hearing warrants close monitoring and appropriate intervention^11,12^.

Unilateral hearing loss in early development promotes reorganization along the auditory pathways which is often difficult to reverse if treatment to provide bilateral input is delayed^11,13^. Early evidence came from both cats and children with bilateral deafness who received a unilateral CI. Strengthening of pathways from the hearing ear was found in the auditory brainstem^14,15^ and in auditory cortices^13^. The cortical preference for the stimulated ear persisted in children who received bilateral CIs with delays of > 2–3 years^16^. The asymmetry of auditory input in these cohorts is similar to that experienced by children with SSD^17^. More mild asymmetries from experimentally induced unilateral conductive hearing loss caused similar neuroplastic changes in young rats^18,19^ and impaired sound localization in young ferrets^1^.

The age at onset of unilateral hearing loss could affect the degree of neural reorganization. For example, aural preference for the hearing ear was clearer in bilaterally deaf cats who received a unilateral CI at younger rather than older ages^20^. Similarly, effects of experimentally induced conductive hearing loss in one ear decrease as animals age^18,19,21^. It is possible that the neuroplastic response to unilateral hearing loss is different after cytoarchitectural maturation of the auditory system^22^. However, adults with SSD also experience strengthening of cortical responses from the intact ear^23–26^. Although cortical reorganization has been shown in young children with SSD^17^, there has been a gap in knowledge related to the possibility of such changes in older children and adolescents with sudden late onset of SSD.

CIs have only been provided to a small number of children with SSD globally. These children provide a unique opportunity to compare the relative strengths of responses to stimulation of hearing and deprived ears, as well as to examine their auditory development. CI-induced plasticity to promote bilateral hearing has been shown in other paediatric cohorts. Bilateral CIs promoted expected cortical representation from both ears when provided to young children with limited delay^11,16^ and similar findings were revealed in children who had asymmetric hearing loss and received a CI in their deaf ear^27^. More recently, longitudinal measures in a small group of toddlers with SSD demonstrated the potential for the CI to reverse cortical preference for the intact ear over a short period of 6 months^17^. However, CIs in children with SSD are not considered standard of care at present in most countries^28,29^. Even in adult patients with SSD, CIs are more often considered to alleviate incapacitating tinnitus in the deaf ear ^30,31^. Benefits in these rare cohorts include better speech perception in noise, improved sound localization, and alleviation of tinnitus^31–35^. Recent findings in small cohorts of children with SSD who have received CIs suggest improved speech perception in quiet and noise ^29,36–39^. Moreover, many children in these cohorts wear their devices consistently ^37,39–41^. Such benefits rely on establishing neural representation from the deaf ear and decreasing preference for the intact ear to promote bilateral auditory development through CI use. These issues were examined in the present study.

Our hypotheses were that: (1) bilateral hearing in early life does not protect children from reorganization in auditory cortex after later onset of SSD and (2) effects of short duration SSD in children can be reversed by chronic and consistent CI use. In this study, auditory cortical responses were evoked with acoustic sound to the normal hearing ear and with electrical stimulation from the CI of the deaf ear and recorded in children with early- (n = 15) and late-onset (n = 7) SSD. Cortical responses measured at initial (0–1 month) CI use were compared those acquired repeatedly after chronic CI use (> 3 months).

## Results

### Initial response peaks mark activity in auditory cortices

**Figure 1:**
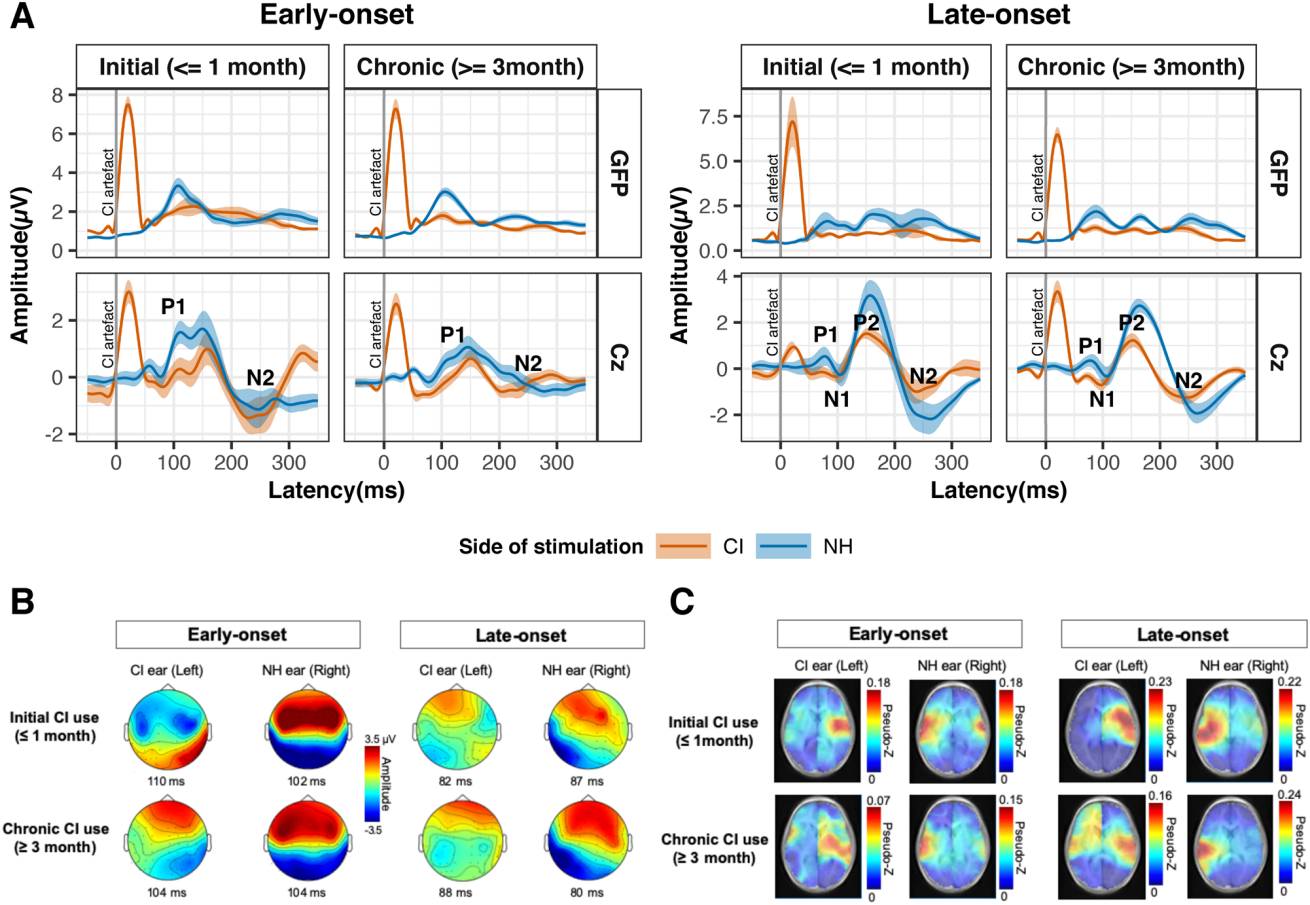
Auditory-evoked surface and source cortical activity. **(A)** The global field power (GFP; spatial standard deviation of surface potentials across electrodes) and cortical response at Cz electrode were averaged per group at two stages of initial cochlear implant (CI) use (0–1 month, early-onset: 15 responses in 11 children; late-onset: 6 responses in 6 children) and chronic CI use (≥ 3 month, early-onset: 27 responses in 15 children; late-onset: 14 responses in 6 children). Mean (solid line) ± standard error (shaded region) amplitude was plotted as a function of post-stimulus time for each stimulation mode. **(B)** Topographic distribution of mean average-referenced EEG activity across the surface of the head at the peak latency. **(C)** Axial view of mean evoked source activity (post-omnibus pseudo-Z) in ~ 64 k voxels that were evaluated using the TRACS beamformer shows the highest activation in the auditory cortex contralateral to the stimulated ear (normal hearing (NH) or cochlear implant (CI)). Note: The images in **(B)** and **(C)** were generated by flipping the x-axis in right CI users before averaging for display.

The group mean of global field power across all recording electrodes and the grand-mean averaged response at the Cz electrode evoked by electrical stimulation in the CI ear and acoustic stimulation in the normal hearing (NH) ear are shown for the early- and late-onset SSD groups at both initial and chronic stages of CI use in Fig. 1A. The younger early-onset group had immature responses characterized by a one positive peak at 100–200 ms (P1) followed by a large negative peak (N2) (leftward plots). The rightward plots in Fig. 1A show the same responses in the older late-onset group, with a small emerging adult-like P1-N1-P2 peak complex followed by a clear N2 peak^42^. Average-referenced topographic maps of surface EEG activity at the group averaged P1 latencies are shown in Fig. 1B. These plots indicate that stimulation in the NH ear evoked positive activity at frontal electrodes in both groups at both recording periods. This was also true of responses evoked by the CI in the late-onset group at both times. By contrast, the early-onset group showed a broad frontal negativity coupled with posterior positivity in response to initial CI stimulation. After chronic CI use, this abnormal topography reversed and expected frontal positive activation was measured, consistent with our previous finding in a subgroup^17^. Figure 1C plots the mean pseudo-Z values of sources underlying the P1 peaks in all children. Largest signals (shown by hot colours) are evident in the left and right auditory cortices with clearest activation in the cortices contralateral to the stimulated ear.

### Aural preference for the normal hearing ear is evident at initial CI activation in children with both early- and late-onset single sided deafness

During the first month of CI use, EEGs were recorded in 11 children with early-onset SSD (4 recorded twice = 15 recordings) and in six children with late-onset SSD. In eight recordings, data analysis was restricted to one condition: stimulation from only the NH ear (early-onset group: n = 4; late-onset group: n = 3) or from only the CI (1 in early-onset group). This occurred either because testing could not be completed or because the response peaks did not meet the signal to noise criteria described in the methods.

**Figure 2:**
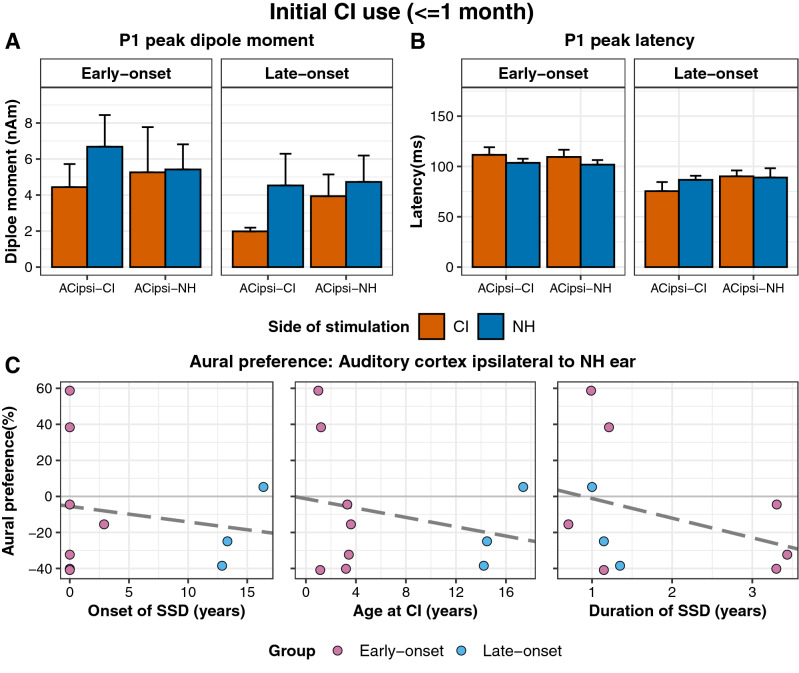
P1 measures and aural preference at initial CI use. **(A) **Mean (± 1 SE) peak dipole moments in auditory cortices at initial (≤ 1 month) stage of CI activation reveal a trend toward larger responses to the NH than CI ear in both auditory cortices in the late-onset group (p = 0.06, 6 responses in 6 children) but not in the early-onset group (p = 0.81, 15 responses in 11 children). **(B)** Corresponding mean (± 1 SE) latencies of peak dipoles show faster responses in the late than early-onset group (p < 0.01). **(C)** Abnormal (grey area) ipsilateral aural preference was measured in 7 of 10 children at this initial stage which could not be significantly predicted by the onset of SSD (p > 0.05), the age at CI (p > 0.05), or the duration of SSD (p > 0.05). ACipsi-CI: Auditory cortex ipsilateral to the CI ear; ACipsi-NH: Auditory cortex ipsilateral to the NH ear. Note: overlapping data points (− 40.86 and − 40.18%) from two children with congenital onset of SSD (0 years) in C-left plot.

Mean dipole moments in both auditory cortices and related latencies in all available responses are shown for both stimulus conditions (NH and CI) at initial CI activation in Fig. 2A,B. In the early-onset group, dipole strength, measured as dipole moment (nAm), was not significantly different between the left and right auditory cortices or ear stimulated (ear stimulated: *F*(1, 41.91) = 0.22, p = 0.64; side of cortex: *F*(1, 39.73) = 0.06, p = 0.81; ear stimulated × side of cortex: *F*(1, 39.73) = 1.30, p = 0.26). In the late-onset group, dipole moments tended to be stronger for responses evoked by stimulation to the NH ear than responses evoked by CI stimulation, but were not different between cortices (ear stimulated: *F*(1,12.9) = 4.30, p = 0.06; side of cortex: *F*(1, 11.52) = 1.25, p = 0.29; ear stimulated × side of cortex: *F*(1, 11.52) = 0.84, p = 0.38). As shown in Fig. 2B, these sources occurred at shorter latencies in the late-onset group than in the early-onset group but no significant latency differences by side of cortex or ear stimulated were found (group: *F*(1, 62) = 12.21, p < 0.01; side of cortex: *F*(1, 62) = 0.29, p = 0.59; ear stimulated: *F*(1,62) = 0.05, p = 0.82). Aural preference measures, which require complete data from both ears, were available from this time period in 10 children (early-onset group = 7, late-onset group = 3). Data from the cortex ipsilateral to the NH ear are shown in Fig. 2C; 7 of the 10 children (early-onset = 5/7 and late-onset = 2/3) demonstrated abnormal preference for the ipsilateral NH ear at this initial stage of CI use. The degree of abnormal aural preference measure could not be predicted by the age at onset of SSD (*F*(1,8) = 0.24, p = 0.64), age at CI (*F*(1,8) = 0.49, p = 0.51), or the duration of SSD (*F*(1,8) = 1.12, p = 0.32).

### Abnormal aural preference for the normal hearing ear is not completely reversed with CI use

**Figure 3:**
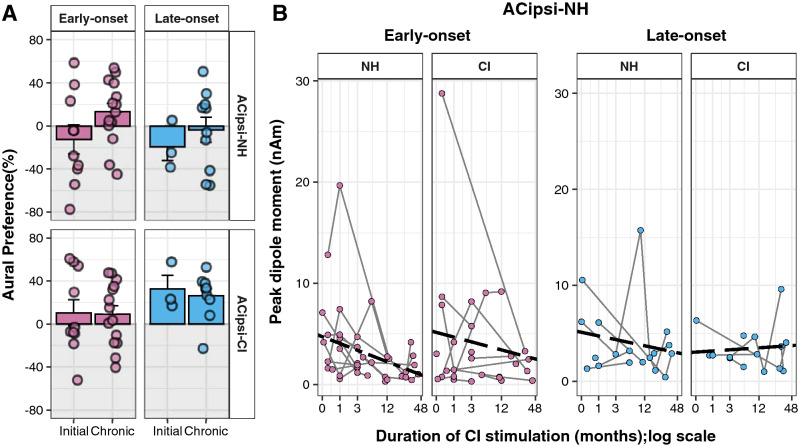
Changes in auditory cortical activity from initial to chronic CI use. **(A)** Aural preference for each response/child (dots) and mean (± 1 SE) data (bars) quantify the overall change between initial (early-onset: 10 responses in 7 children; late-onset: 3 responses in 3 children) and chronic (early-onset: 14 responses in 11 children; late-onset: 10 responses in 6 children) periods of CI use, indicating, in both groups, a tendency toward normal aural preference in the AC ipsilateral to the NH ear (p = 0.07) and maintained representation of the NH ear in the opposite cortex in both groups (p > 0.05). **(B)** Dipole changes with CI use are shown for each child, revealing a time-dependent reduction of responses stimulated by the NH ear in the ipsilateral cortex in the early-onset group only (p = 0.06). ACipsi-CI: Auditory cortex ipsilateral to the implanted ear; ACipsi-NH: Auditory cortex ipsilateral to the NH ear.

Cortical responses were also measured after chronic (≥ 3 month) CI use. The Supplementary Fig. 1 shows the mean (± 1SE) dipole moments and latencies in the early and late-onset groups at this later time period. Mean aural preference data for both groups at initial CI use and after chronic CI are shown in Fig. 3A. Aural preference for the NH ear in the ipsilateral cortex (top panel) tended to diminish between these periods (period: *t*(37) = 1.84, p = 0.07; group: *t*(37) = -0.31, p = 0.76; period × group : *t*(37) = − 0.38, p = 0.71). Despite the variability, there still is a marked change with chronic CI use. The proportion of children with normal aural preference in the chronic period was 8 of 11 (73%) in the early-onset group and 3 of 6 (50%) in the late-onset group. Further testing revealed that the larger change in aural preference from initial to chronic CI use occurred in the early-onset group (Initial-Chronic estimate: − 25.6 (p = 0.09) in early-onset and − 15.7 (p = 0.53) in late-onset). In the opposite cortex, the expected contralateral preference for the NH ear was maintained in both groups (bottom panel) and tended to be stronger in the late vs early-onset group (period: *F*(1, 31.40) = 0.36, p = 0.55; group: *F*(1, 22.54) = 3.07, p = 0.09; period × group: *F*(1, 31.40) = 0.19, p = 0.67).

Changes in dipole moments evoked in each stimulus condition were assessed in the measures taken repeatedly with ongoing CI use to further understand the changes in aural preference in the cortex ipsilateral to the NH ear. Dipole moments tended to differ by mode of stimulation and also to interact with group and side of cortex (group: *F*(1,22.68) = 0.01, p = 0.91; side of cortex: *F*(1,178.11) = 0.11, p = 0.74; ear stimulated: *F*(1,181.15) = 3.57, p = 0.06; time of CI use: *F*(1,24.73) = 1.34, p = 0.26; group × ear stimulated: *F*(1,181.15) = 2.92, p = 0.09; side of cortex × ear stimulated: F(1,178.11) = 3.22, p = 0.07). Based on these results, dipole change with time of CI use was further investigated in each group, in each cortex, to each ear of stimulation separately. As shown in Fig. 3B, dipole moments evoked by the NH ear in the ipsilateral cortex tended to decrease in the early-onset group with CI use (*F*(1,16.49) = 4.15, p = 0.06) but were maintained in the same cortex with CI stimulation of the contralateral ear (*F*(1,11.25) = 0.53, p = 0.48). The dipole moments by NH stimulation in the ipsilateral cortex in the early-onset group after chronic stimulation are also shown in the Supplementary Fig. 1. Dipole moments in the late-onset group reduced in the 3 of 6 children who had initial time points (0–1 month), when evoked by the NH ear in the ipsilateral cortex and there was no significant dipole moment change in this group with CI use (CI use: with NH stimulation: *F*(1,18) = 0.67, p = 0.42; with CI stimulation: *F*(1,5.04) = 0.09, p = 0.78).

Because aural preference relies on data from both stimulus conditions at each test time, repeated measures of aural preference were only possible to calculate in 6 children in the early-onset group and 3 children in the late-onset group. With limited power, the changes in aural preference across time in the cortex ipsilateral to the NH ear were not significant in either the early-onset (*F*(1,17.45) = 0.27, p = 0.61, intercept = − 2.26, slope = 7.11) or late-onset groups (*F*(1, 13) = 0.84, p = 0.38, intercept = − 22.15, slope = 15.73). Similarly, no significant changes in aural preference were found with CI use on the opposite cortex in both the early-onset group (CI use: *F*(1,15.8) = 0.43, p = 0.52) and the late-onset group (CI use: *F*(1,13) = 2.11, p = 0.17).

### Cortical representation of normal hearing ear recovers in children with late-onset SSD but representation from the deaf ear weakens

**Figure 4:**
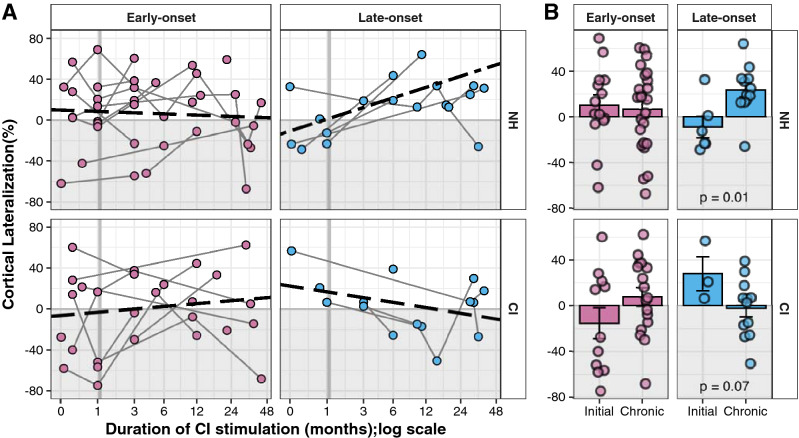
Changes in cortical lateralization with CI use. **(A)** Cortical lateralization to stimulation of each ear is plotted for individual children with duration of CI use (early-onset NH ear: 38 responses in 15 children; late-onset NH: 18 responses in 6 children; early-onset CI ear: 28 responses in 15 children; late-onset CI: 15 responses in 6 children). Values for both ears show variability which is maintained over time in the early-onset group. The late-onset group shows abnormally strong responses in ipsilateral cortex (-values) to NH stimulation initially which reverse with CI use (*F*(1, 6.71) = 4.49, p = 0.07). However, expected contralateral lateralization to CI stimulation (+ values) measured at initial CI use is lost with chronic CI experience (*F*(1, 13.68) = 2.28, p = 0.15). **(B)** Mean (± 1 SE) cortical lateralization data quantify the overall change between initial and chronic periods of CI use, indicating no significant changes in the early-onset group (p > 0.05) but significant shifts in the late-onset group that depends on the ear stimulated (interaction ear x period: *F*(1,33) = 11.88, p < 0.01).

Cortical responses to stimulation in each ear were assessed for expected increases in the cortex contralateral to the stimulation compared to the ipsilateral cortex using the cortical lateralization measure. Data from individual children are shown in Fig. 4A. In the early-onset group, cortical lateralization from both the NH ear and from the CI showed high variability. Over time of CI use, contralateral lateralization from the NH ear reduced (negative slopes) in 11 of 14 children. Of note is that these reductions were small and thus lateralization did not shift into abnormal values (i.e. expected contralateral lateralization was maintained from the NH ear). Overall, there was no significant change in cortical lateralization from the NH ear stimulation with CI use in the early-onset group (CI use: *F*(1, 10.45) = 0.16, p = 0.69). These findings are consistent with the tendency for dipoles evoked by NH stimulation to decrease with CI use in both the ipsilateral cortex as discussed in “Consistent device use helps the auditory pathways to develop in the early-onset group and to recover from SSD in the late-onset group” (CI use: *F*(1,16.49) = 4.15, p = 0.06) and the contralateral cortex (CI use: *F*(1,15.26) = 3.47, p = 0.08).

From the CI ear, children in the early-onset group showed variable changes in cortical lateralization with CI use; 8 of 13 had positive slopes indicating a shift toward more expected contralateral lateralization of responses from the CI ear. Overall, there was no significant effect of CI use on cortical lateralization from the CI ear in the early-onset group (CI use: *F*(1, 8.90) = 0.35, p = 0.57). Summarized data at initial and chronic CI use, shown in Fig. 4B, confirms that there were no significant changes in cortical lateralization from either the NH or CI ear (ear stimulated: *F*(1,66) = 1.76, p = 0.19; period: *F*(1,66) = 1.13, p = 0.29, ear × period: *F*(1,66) = 2.12, p = 0.15). Moreover, analysis of dipoles evoked by CI stimulation reveal no significant changes with CI use in either the ipsilateral (time of CI use: *F*(1,12.03) = 1.04, p = 0.33) or contralateral cortex (time of CI use: *F*(1,11.25) = 11.25, p = 0.48).

In the late-onset group, cortical lateralization from the NH ear shifted from abnormal to expected values (positive slopes in 6 of 7 children). As shown in Fig. 4A, there was a trend for this reversal to occur across the children in the group (CI use: *F*(1,6.71) = 4.49, p = 0.07). From the CI ear, cortical lateralization shifted from expected contralateral to ipsilateral values (negative slopes) in all 7 children but these changes were not significant across children in the group (CI use: *F*(1,13.68) = 2.28, p = 0.15). Summarized data at both periods of CI use, shown in Fig. 4B, confirms a significant reversal of abnormal representation of the NH ear but a tendency toward abnormal responses in the CI ear (ear stimulated: t(33.0) = − 2.35, p = 0.02; period: t(33) = 2.10, p = 0.04; interaction ear stimulated × period: t(33) = 3.45, p < 0.01, post-hoc NH: Initial-Chronic contrast: estimate = − 32.5, p = 0.01 and CI: Initial-Chronic: estimate = 30.1, p = 0.07). Dipole moment did not significantly change over time in either cortex when evoked by CI stimulation (in the ipsilateral cortex: time of CI use: *F*(1,6.79) = 2.55, p = 0.16; in the contralateral cortex: time of CI use: *F*(1:5.04) = 0.09, p = 0.78) or NH stimulation (in the ipsilateral cortex: time of CI use: *F*(1,18) = 0.67, p = 0.42; in the contralateral cortex: time of CI use: *F*(1,16.92) < 0.01, p = 0.93).

### Expected cortical representation increases with amount of consistent daily CI use

**Figure 5:**
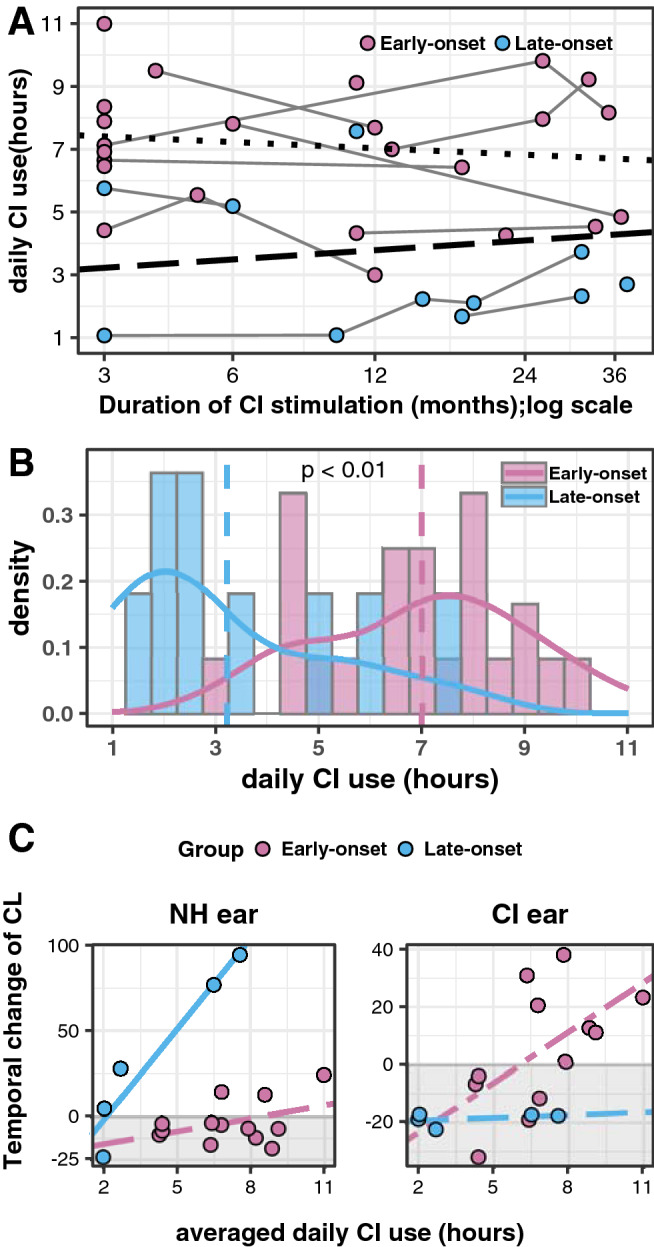
Daily hours of CI use and effects on cortical lateralization. **(A)** Average daily hours of device use with chronic CI use (≥ 3 month) is plotted over time of CI use for each child (14 with early-onset and 5 with late-onset) at each recording time. There is high variability and no significant change over time (p > 0.05). **(B)** The distribution of hours of daily CI use are shown for each group. Average daily CI use was significantly reduced in the late-onset group (p < 0.01). C) The changes of lateralization by the time of CI experience (slopes for individual child from Fig. 4A) was plotted as a function of averaged daily hour of device use. Increased daily device use promotes expected changes toward contralateral lateralization to the NH stimulation in the late-onset group (p = 0.01) and to the CI stimulation in the early-onset group (p = 0.06).

Consistency of device use during the chronic stage of CI use (≥ 3 month) was analyzed using the average hours of CI use per day. These data were extracted from a datalog kept by the CI speech processor. As shown in Fig. 5A, the average hours of device use per day varied from 1 to 11 h and was significantly longer in early-onset group (mean ± s.d., range, median: 6.99 ± 2.04 h, 3.00–10.99 h, 7.06 h) than late-onset group (mean ± s.d., range, median: 3.22 ± 2.11 h, 1.07–7.57 h, 2.32 h) but did not change over time (Fig. 5A, group: *F*(1,32.30) = 6.76, p = 0.01; time: *F*(1,22.08) = 0.05, p = 0.82, group × time: *F*(1,22.08) = 1.36, p = 0.26). The distribution of daily CI use, plotted in Fig. 5B, further reveals the differences in the distribution of daily CI use between the two groups (two-sample Kolmogorov–Smirnov test, D = 0.69, p < 0.01).

The effect of consistent daily CI use on the change in cortical lateralization over the period of chronic CI use per child was assessed. Cortical lateralization over the log of time, as shown in Fig. 4A, was extracted from the associated mixed model for each child. Negative values indicated changes away from expected contralateral lateralization and positive values indicated changes toward the expected contralateral lateralization with CI use. These values are plotted by the amount of daily CI use in Fig. 5C. Cortical lateralization measures were significantly affected by daily CI use and group; both of these factors interacted with ear stimulated (daily CI use: F(1) = 11.95, p < 0.01; group: F(1) = 14.82, p < 0.01; ear stimulated: F(1) = 3.31, p = 0.08; daily CI use × group × ear stimulated: F(1) = 17.98, p < 0.01).

The effect of daily CI use on changes to cortical lateralization were further analyzed in each group separately. In the early-onset group, larger positive changes (toward expected contralateral lateralization) from the CI ear occurred in children who used their CI for more hours in the day (CI ear: *F*(1,10) = 4.731, p = 0.06). The slight reduction in cortical lateralization from the NH ear, as shown in Fig. 4A, was not significantly affected by daily CI use (CI daily use: *F*(1,11) = 2.156, p = 0.17). In the late-onset group, an effect was found in responses from the NH ear; expected changes toward contralateral lateralization (positive values) from the NH ear, as shown in Fig. 4, were larger with longer daily CI use (Daily CI use: *F*(1,3) = 32.87, p = 0.01). Although responses from the CI ear tended to decline toward abnormal cortical lateralization (Fig. 4), these changes were not affected by duration of daily CI use in the late-onset group: (daily CI use: *F*(1,3) = 0.57, p = 0.51).

## Discussion

### SSD allows preference for the better hearing ear to emerge in children

Abnormal aural preference for the NH ear was shown in a large proportion of children in the present cohort (7 of 10 children; Fig. 2) at initial CI use as measured by stronger dipole strength in response to stimulation from the NH ear than from the ear with CI (Figs. 2, 3) as well as an expected lateralization of cortical responses from the NH ear to the contralateral cortex (Fig. 4). This finding corroborates evidence that asymmetric hearing in early life promotes enhanced representation of the better (or only) hearing ear in the auditory cortices (for review, Gordon and Kral 2019)^13^. Strengthened cortical responses to the better hearing ear have been observed in animal models with conductive hearing loss^18,19^ and profound sensory/neural hearing loss^20,43^. Abnormal preference for the better hearing ear also develops in young children with asymmetric hearing and is more difficult to reverse as the duration of asymmetric hearing lengthens^16,44^. The concern is that preference for one ear limits functional use of the poorer ear^16,27,36^ and disrupts binaural hearing^45,46^.

In the present cohort of children with early-onset SSD, representation of the NH ear in the ipsilateral cortex was shown in Figs. 2 and 3; responses in this cortex had higher dipole moments in response to the NH ear than the CI at initial CI use in five of seven children. As shown in Fig. 4, expected lateralization from the NH ear to the contralateral cortex was maintained at initial CI use in five of ten children and only two of ten children showed clearly abnormal ipsilateral lateralization (values: − 61.77% and − 42.12%). Similar results in a group of children with bilateral deafness who had used a unilateral CI from young ages suggest that monaural stimulation from either a normal hearing ear or a cochlear implant in early development, without input from the opposite ear, allows uninhibited strengthening from the stimulated side to both cortices^16^. Meanwhile, the deprived pathways retain rudimentary organization from the ear to the cortex, likely mediated by genetic programming, but are left naïve^14,43,47^. Initial CI stimulation of these deprived pathways can evoke abnormal responses. As shown in Fig. 4, responses to initial CI input lateralized to the ipsilateral cortex in 5 of 8 children, reflecting a relatively weaker input from the implanted ear to the contralateral cortex (ipsilateral to NH ear).

As shown in Figs. 2 and 3, two of seven young children in the early-onset group (Child 1 and 4) did not show aural preference for the NH ear at initial CI use. These children were amongst the youngest of our cohort and thus had more limited durations of monaural experience which might have been too short to override stimulus-independent development. Moreover, the duration of SSD may or may include the pre-natal period and this variability is likely to be dependent on etiology. Indeed, Child 1 had congenital CMV which causes progressive hearing loss with variable onset ^48^; it is therefore possible that this child had early in utero access to sound.

A novel finding in the present cohort was that, as shown in Fig. 2, a short period of SSD was sufficient to result in aural preference for the NH ear in two of three children who acquired SSD in later childhood/adolescence (Child 18 and 19). Results in the other child (Child 22) with the shortest duration of SSD were also abnormal with no clear preference for either ear. Despite only 1.17 mean years of SSD in these three children, responses in the auditory cortex ipsilateral to the NH ear were larger when evoked by the NH ear than the CI ear at initial CI use. As also shown in Fig. 2, there were no effects of age at test or age at onset on the degree of aural preference for the NH ear. Further, there was no effect of duration of SSD, perhaps reflecting the narrow range (< 4 years) permitted in the present cohort. These findings are consistent with evidence from animals with experimentally induced hearing loss in one ear at different ages. Strengthened responses in the ipsilateral cortex from the intact ear have been found in both juvenile-onset and adult-onset SSD, though the effect size reduces with age^18,19,21^. Similarly, in both adults and children with SSD, imaging studies demonstrate increased ipsilateral cortical responses to the intact ear^17,23–26^.

The rapid change in cortical responses after a sudden change in hearing in adults is consistent with behavioral adaptations to unilateral hearing. Adults who experience even a short period of monaural plugging show improvements in sound localization^49,50^. Importantly, behavior can reverse to normal once the plug is removed^50,51^ but this is particular to adults^52,53^. Ear plugging in early stages of development promote changes which are more difficult to reverse^54^, suggesting that early and late-onset hearing loss in one ear are mediated differently. Whereas the immature auditory pathway may experience architectural change, including neuronal and glial loss in brainstem structures connected to the deaf ear^55,56^, the mature pathway responds to the loss of input from one ear through changes of inhibitory/excitatory transmission^57–60^. In the adolescents, SSD onset may be occurring during a potentially important development transition, reflected by maturation of cortical responses by ~ 12 years of age^24,47^. Differences in age-related plasticity also explain why providing unilateral CIs in older congenitally deaf white cats does not promote the same cortical preference for the stimulated ear as when the implant is provided at earlier developmental stages^20^.

In sum, data from the present study indicate that unilateral hearing occurring after normal binaural development and cortical development driven by hearing from one ear both promote cortical preference for the hearing ear.

### Recovery from cortical effects of SSD are variable in children using CIs

Data shown in Fig. 3 suggest that reversal of aural preference for the NH ear can be challenging in children. In the early-onset group, dipoles in the cortex ipsilateral to the NH ear decrease in strength in response to stimulation of the NH ear but are maintained from the CI. This explains the slight shift in mean aural preference from -12.50% to 13.08% between initial CI use and later (chronic) use with marginal significance (p = 0.07). Whereas 5 of 7 children in this group showed abnormal ipsilateral preference at initial CI activation, expected contralateral preference with CI use was found in 8 of 11 children with early-onset SSD. Similar shifts were previously reported in a small subset of this group over the first 6 months of CI use^17^. In the present cohort, no significant changes in cortical lateralization from either ear were found with implant use although 7 of 12 children either retained or gained representation of the CI in the contralateral cortex with a small shift of mean cortical lateralization from -15.24% to 7.70% from initial CI activation to chronic CI use (Fig. 4).

The goal of providing CI in children with early-onset SSD is to provide bilateral input and promote expected representation from both ears during important early stages of development. This was realized in a group of children who were deaf from infancy and received bilateral CIs simultaneously^16^. Symmetric bilateral input can also be promoted with bimodal hearing in children who use a hearing aid in one ear and a CI in the other ear but this depends on early access to bilateral hearing and residual hearing^27,36^. When residual hearing in the hearing aid ear is poor, the CI becomes the preferred ear and when there is significant residual hearing, preference for the better hearing ear may be maintained. Thus, the findings that aural preference for the NH ear in the present cohort of children with early onset-SSD can be difficult to reverse in some children, reflects the reality that sound is better accessed through the NH ear than from the CI.

The children in the late-onset group showed little change in aural preference for the NH ear in the ipsilateral cortex from initial to chronic CI use. As shown in Fig. 3, the many more recordings from this group available in the chronic CI use stage reveal variability; preference for the NH ear persisted in three of six children in this group. Analyses of dipole strength with CI use revealed small reductions in input from the NH ear but these were not statistically significant across the cortices. Data from the opposite cortex, also shown in Fig. 3, indicate that these children maintain a clear representation of the NH ear in the contralateral cortex at both initial and chronic stages of CI use. On the other hand, children in the late-onset group experienced larger changes in cortical lateralization as shown in Fig. 4. Input from the NH ear shifted from being weighted to the ipsilateral cortex to having expected contralateral lateralization. Input from the CI, however, showed a tendency to deteriorate from contralateral lateralization to more equal weighting of input in both cortices. This occurred in four of six children. Overall, these findings suggest a weakening of representation from the deaf ear despite cochlear implantation in children with late-onset deafness.

### Consistent device use helps the auditory pathways to develop in the early-onset group and to recover from SSD in the late-onset group

We questioned whether the challenges to reversing aural preference for the NH ear in both groups and the tendency for deterioration of cortical representation from the deaf ear in the late-onset SSD group might be explained, in part, by consistency of device use. The average daily CI use, monitored in each child’s CI speech processor, varied from 1 to 11 h and there was no significant change over time as shown in Fig. 5. This was consistent with data reported previously from our group^41^. In the present cohort, daily CI use was significantly shorter in the children with late-onset than early-onset SSD by 3.8 h, on average. Importantly, the consistency of device use had significant effects on the degree of change in cortical lateralization by group and side of ear stimulated. As shown in Fig. 5, children with early-onset who used their CIs for longer daily periods achieved more positive change (toward development of normal contra-lateralization) from the CI ear. There was little effect of daily CI use on the NH ear in this group. The data suggest that representation of the CI in the contralateral cortex requires ~ 6.5 h of CI use per day and that the representation of the NH ear is maintained even with many hours of CI stimulation.

In the late-onset group, consistent daily CI use helped to reduce abnormal ipsilateral lateralization from the NH ear and to restore expected contralateral lateralization (Fig. 5) but could not stop negative change (toward ipsilateral lateralization) from the implanted deafened ear. These findings suggest that a few hours of CI use a day in children who experience late-onset SSD, helps to support the NH ear by limiting the need for strengthening in the ipsilateral cortex. At the same time, this relatively limited duration of input from the CI each day may be insufficient to maintain the pathways from the deafened ear. Adult-onset deafness also results in deterioration of the auditory pathways including changes in functional and structural cortical connectivity^61–63^ and deterioration in white matter^61^. The inability of CI use to halt such changes in the children/adolescents with late-onset SSD during an important period of auditory maturation, when listening and cognitive demands are high, could also be due to limitations in CI stimulation in the deaf ear and along the bilateral pathways. Cls provide more limited access to the spectrotemporal features of acoustic sound than normal^64,65^ and bimodal input (CI in one ear, acoustic hearing in the other) mismatches interaural place, level and timing of stimulation^66–68^ which impairs binaural function^69^.

## Summary and conclusions

In the present study, cortical plasticity was compared in a group of children who experienced SSD in early life and in a group who lost hearing in one ear in later childhood/adolescence. Aural preference for the hearing ear was measured in both groups at initial CI use, confirming that cortical reorganization driven by unilateral hearing can occur throughout childhood and adolescence. CI use decreased response to stimulation from the hearing ear in both groups, suggesting diminishing reliance on this ear alone. This was clearest for children in the late-onset group who used their CI for the greatest number of hours daily. Ongoing CI use promoted strengthening of pathways from the deprived ear most clearly in children in the early-onset group who used their CI consistently but did not protect children with late-onset SSD from ongoing deterioration of pathways from the deaf ear.

## Methods

### Participants

Twenty-two children with SSD who received a CI in their deaf ear participated in this study. Parental written informed consent was obtained for all participants according to study protocol #1000002954 approved by the Hospital for Sick Children Research Ethics Board, and all methods were carried out in accordance with relevant guidelines and regulations. Pre-CI pure tone average (0.5, 1, 2, and 4 kHz) of hearing threshold was 97 ± 18 dB HL (range: 68–120 dB HL) in the deaf ear and 14 ± 8 dB HL (range: 0–30 dB HL) in the other normal hearing (NH) ear. Demographic details are provided for each child in Table 1. Children were divided into two groups based on their age at onset of SSD. A clear group of seven children who experienced a sudden onset of SSD in adolescence (> 10 years old) were defined as “late-onset” SSD. Another 13 children all experienced SSD either from birth and two children (ID: 14 and 15) experienced SSD related to cCMV during early years of auditory and language development (3.0 and 4.9 years of age)^22,70^ and were thus classified as “early-onset”. Effects of age at onset were included in statistical analyses. Ten of fifteen children in the early-onset group and 5 of 7 in the late-onset group had severe-to-profound hearing loss in their left ear. In the early-onset group, congenital cytomegalovirus (cCMV) infection (n = 10) was the most common cause of SSD, followed by inner ear malformation (n = 3). In the late-onset group, SSD developed suddenly following idiopathic sudden sensorineural hearing loss (n = 3), temporal bone fracture (n = 2) or acoustic trauma (n = 2). Children received a CI in the deaf ear at mean (SD) age of 2.9 (1.6) years (early-onset group) and 14.4 (2.0) years (late-onset group) after a restricted period of single-sided hearing (mean (SD) = 2.4 (1.5) years in early-onset group and 1.4 (0.3) years in late-onset group). Electroencephalographic (EEG) data from the first 6 months of CI use in Children 3, 8, 10, 11, and 14 have been previously reported ^17^.

**Table 1:** Patient demographics.

Child	Implanted ear	Etiology	Age at SSD onset (years)	Age at CI (years)	Duration of SSD (years)
**Early-onset SSD group**					
1	Left	cCMV	0	1.0	1.0
2	Left	IEM	0	1.1	1.1
3	Left	cCMV	0	1.2	1.2
4	Right	Unknown	0	1.2	1.2
5	Left	cCMV	0	1.8	1.8
6	Left	IEM	0	2.1	2.1
7	Left	cCMV	0	2.6	2.6
8	Left	cCMV	0	3.2	3.2
9	Right	Meningitis	0.6	3.3	2.7
10	Left	cCMV	0	3.3	3.3
11	Left	IEM	0	3.4	3.4
12	Right	cCMV	0	4.2	4.2
13	Right	cCMV, ear canal atresia in deaf ear	0	5.9	5.9
14	Left	cCMV	3.0	3.6	0.6
15	Right	cCMV	4.9	6.1	1.2
	Mean ± s.d.		0.6 ± 1.4	2.9 ± 1.6	2.4 ± 1.5
**Late-onset SSD group**					
16	Left	SSNHL	10.3	11.8	1.5
17	Left	TB fracture	10.8	12.2	1.4
18	Left	SSNHL	12.9	14.2	1.4
19	Left	SSNHL	13.3	14.5	1.2
20	Right	Acoustic trauma	13.3	14.7	1.5
21	Left	TB fracture	14.2	16.0	1.7
22	Right	Acoustic trauma	16.4	17.4	1.0
	Mean ± s.d.		13.0 ± 2.1	14.4 ± 2.0	1.4 ± 0.3

*SSD* single-sided deafness, *cCMV* congenital cytomegalovirus, *IEM* inner ear malformation, *SSNHL* sudden sensorineural hearing loss, *TB fracture* temporal bone fracture.

Daily CI use was confirmed by datalogs available from the CI speech processor. Datalogs for each child were collected by the clinical audiologist on the same day as EEG recording. On average, datalogs after chronic (≥ 3 month) CI use provided mean daily CI use over a mean of 146.71 days (SD = 106.64), equivalent to the number of days since the previous clinical audiology appointment. Datalogs were available for all children at least once post-CI and repeated datalogs were collected in 14 of the 22 participants.

### Electroencephalography

**Figure 6:**
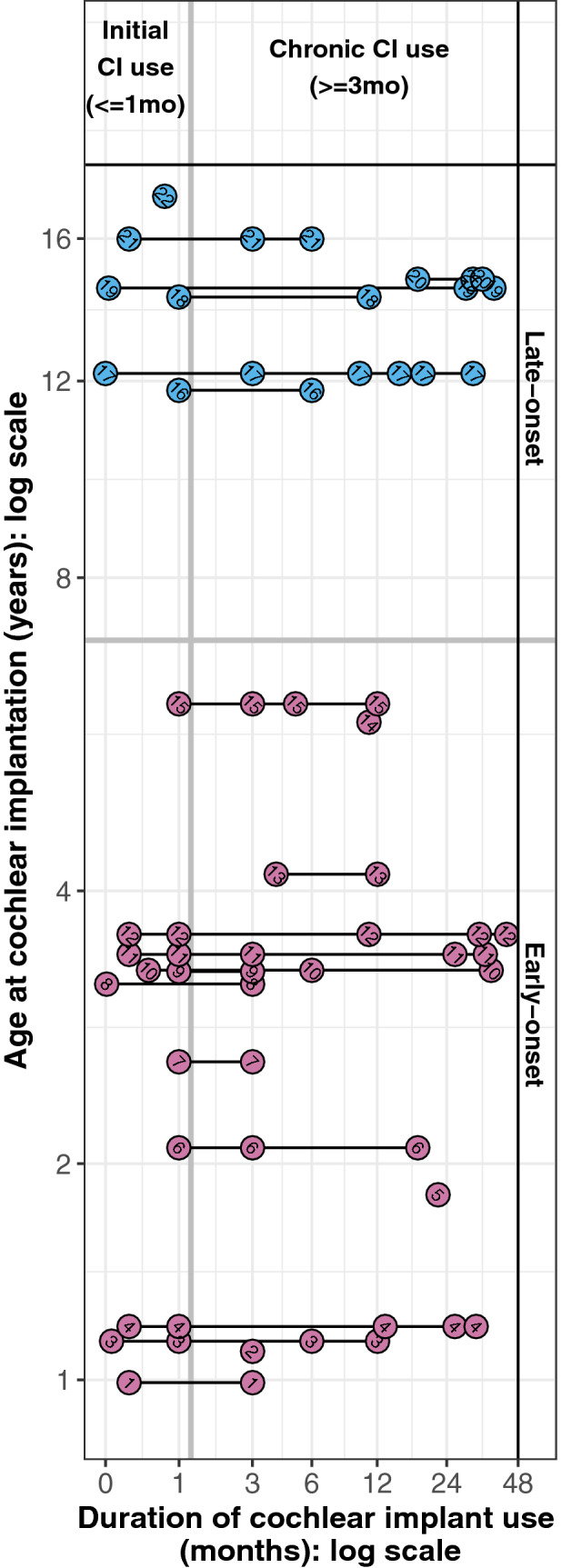
Timing of EEG recording in each child. Children in both the early (pink) and late (blue) onset SSD groups completed multiple EEG recording sessions, spanning from initial CI use (0–1 month) to chronic CI use (≥ 3 month). Study codes (details in Table 1) are shown.

Cortical responses were evoked by acoustic clicks (100 µs) delivered at 250 Hz in trains of 36 ms via an insert earphone to the normal hearing ear or electric biphasic pulses (57 µs pulse-width) delivered at 250 Hz from an apical electrode (#20) of the CI. These stimuli were presented unilaterally at 1 Hz. Stimulation level was based on amplitudes of the auditory brainstem responses as detailed previously^16,66^. Evoked EEG signals were recorded by 62 cephalic electrodes in standard positions detailed previously^16,44,47^. Continuous EEG data were separated into 1000 ms epochs which included a 200 ms pre-stimulus interval. At least 250 epochs were recorded for each condition (CI ear and NH ear stimulation). As shown in Fig. 6, EEG recordings were repeated from the day of CI device activation up to 4 years after surgery. Across the 22 children, a total of 62 recordings were completed; 37 recordings included both a CI condition and NH condition and the remaining 25 recordings included only one condition (CI or NH ear). In 16 children, recordings were completed during initial CI use (0–1 month) and after chronic CI use (≥ 3 month). Initial recordings were not available in 5 children.

### Data analysis

The Time-Restricted Artefact and Coherence Source Suppression (TRACS) method was used to localize source activity in the time window encompassing the first amplitude peak of the surface waveform (Fig. 1A)^16,71^. TRACS is an adaptive spatial filter (linearly constrained minimum variance type beamformer) that estimates the contribution (dipole activity) of each of the 3 × 3 × 3 mm voxels of the brain’s volume to the recorded surface activity, after suppressing 97% of the CI artefact. Because both auditory cortices could have coherent activity, source activity in each hemisphere was evaluated while suppressing activity in the contralateral hemisphere. Age-dependent head geometry and conductivities of the brain, skull and scalp were accounted for by a 3-layer boundary element model that was constructed from age-appropriate Montreal Neurologic Institute magnetic resonance imaging templates generated with the Template-O-matic toolbox. Activity in each voxel was normalized relative to the pre-stimulus baseline (− 200 to − 80 ms) using a pseudo-Z statistic (calculated ratio of the mean signal to the standard deviation of the pre-stimulus baseline). A one-tailed omnibus t-test was also conducted in which half of the trials were flipped in polarity to produce a plus-minus average (which removes the time-locked activity) to determine a statistical threshold pseudo-Z of baseline brain activity (omnibus value). This omnibus value was subtracted from the pseudo-Z value in each voxel in order to identify voxels with higher than baseline brain activity. Peak dipole strength and the corresponding latency were extracted from the source time series for all voxels, but for further analyses, the ten voxels with the largest pseudo-z above omnibus threshold were chosen in both the left (x ≤ − 55) and right (x ≥ 55) auditory cortical areas ( − 35 ≤ y ≤ 5; − 10 ≤ z ≤ 20)^16,17,42^. From those highest ten voxels in each auditory cortex, the maximum dipole strength and the corresponding latency were extracted and averaged for further analyses. For children in which stimulation of one ear evoked activity with pseudo-Z value above threshold in one auditory cortex, the peak dipole moment with highest pseudo-Z value (although below omnibus) was chosen in the less-activated hemisphere to calculate normalized indices. In two children in the late-onset group (Child 16 and 22), peak dipole moment was extracted from P2 instead of P1 at initial CI use, as pseudo-Z at P1 was below threshold in both auditory cortices in these cases and there was only a small emerging N1. This recording was not included in the P1 latency analysis.

Two normalized indices were calculated using the averaged dipole moment from each cortex. Cortical lateralization (%) characterized the difference in dipoles between the contralateral and ipsilateral auditory cortices for each ear as defined by: 100 × (contralateral cortex – ipsilateral cortex)/(contralateral cortex + ipsilateral cortex). Thus, positive cortical lateralization values indicate larger contralateral than ipsilateral cortical activity in response to stimulation from one ear. Aural preference (%) by contrast, was defined by 100 × (contralateral ear – ipsilateral ear)/(contralateral ear + ipsilateral ear). Positive aural preference indicates increased activity by the contralateral ear as compared to the ipsilateral ear in one cortical hemisphere and negative values indicate an ipsilateral aural preference.

### Statistical analysis

Statistical analyses were performed with R using the lmerTest and lme4 packages^72–74^. Given the progressive increase in follow-up intervals, duration of CI use at testing was log-transformed. Variability in dipole moment, latency, aural preference, cortical lateralization, and hours of daily CI use were evaluated using linear mixed effects regression with random intercepts for each child. Fixed predictors included: group, time or period of CI use, cortical side, ear of stimulation, and related interactions. Linear regression was used to assess cortical aural preference at the initial stage of CI use for effects of age at SSD, age at CI, or duration of SSD. Mean values were used for any child with multiple measures within the period of initial CI use. Linear regression was also used to determine effects of daily CI use on the change in cortical lateralization over CI use (cortical lateralization (%)/log(days CI use)). ANOVAs and pairwise post-hoc analyses were implemented using the Satterthwaite method to estimate denominator degrees of freedom for t-statistics of the mixed models. Significance was defined at p < 0.05.

## Supplementary Information

Supplementary Figure S1.

## Data Availability

The datasets generated during and/or analysed during the current study are available from the corresponding author on reasonable request.
