# Supplementary material for: Consistent and chronic cochlear implant use partially reverses cortical effects of single sided deafness in children
Source: Sci Rep. 2020 Dec 9;10:21526. doi: 10.1038/s41598-020-78371-6 (PMC7726152; doi:10.1038/s41598-020-78371-6)
Supplement: Supplementary file 1 — Supplementary Figure S1. [file 41598_2020_78371_MOESM1_ESM.pdf]

Supplementary material

Consistent and chronic cochlear implant use partially reverses cortical effects of single sided deafness in children

Hyo-Jeong Lee[1,2,6], Daniel Smieja[1,2], Melissa Jane Polonenko[1,2], Sharon Lynn Cushing[1,3-5], Blake Croll Papsin[1,3-5], Karen Ann Gordon[1-5*]

[1]Archie's Cochlear Implant Laboratory, Hospital for Sick Children, Toronto, Ontario, Canada

[2]Department of Communication Disorders, Hospital for Sick Children, Toronto, Ontario, Canada

[3]Institute of Medical Science, University of Toronto, Toronto, Ontario, Canada

[4]Department of Otolaryngology-Head and Neck Surgery, Hospital for Sick Children, Toronto, Ontario, Canada

[5]Department of Otolaryngology-Head and Neck Surgery, University of Toronto, Toronto, Ontario, Canada

[6]Department of Otorhinolaryngology-Head and Neck Surgery, Hallym University College of Medicine, Chuncheon, Republic of Korea

Running head: Plasticity with cochlear implant use in children deaf in one ear

Dipole moment significantly changes from initial to chronic CI use (period: $F(1,197.39) = 4.77$, $p = 0.03$; group: $F(1,28.17) = 0.02$, $p = 0.88$; side of cortex: $F(1,179.85) <0.01$, $p = 0.94$; ear stimulated: $F(1,188.81) = 1.85$, $p = 0.17$; group x period: $F(1,197.39) = 2.81$, $p = 0.09$; side of cortex x ear stimulated: $F(1,179.85) = 2.94$, $p = 0.09$). Post-hoc testing reveals significant dipole changes from initial to chronic CI use in the early-onset group occurs in both cortices with stimulation from the NH ear (Initial-Chronic contrast estimate: in the ipsilateral cortex: 3.05, $p = 0.02$; in the contralateral cortex: 3.38, $p < 0.01$).

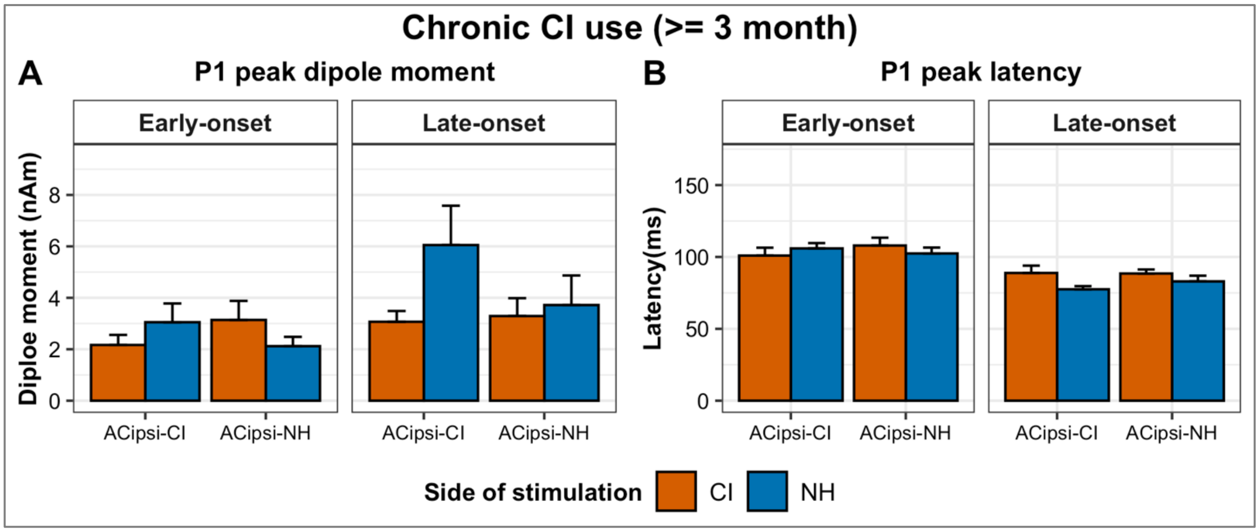

Supplementary Figure S1. In the early-onset group, cortical responses from both ears after chronic CI use are stronger in dipole moment (nAm) to the contralateral than ipsilateral ears as expected normally (side of cortex: $F(1,65.03) <0.01$, $p=0.96$; ear stimulated: $F(1,69.68) =0.08$, $p=0.78$; side of cortex x ear stimulated interaction: $F(1,65.03) =5.27$, $p=0.02$). In the late-onset group, cortical responses are stronger (nAm) to the NH ear than to the CI (ear stimulated: $t(44.81) =2.12$, $p=0.04$; side of cortex: $t(44.50) = 0.87$, ear stimulated x side of cortex: $t(44.50) = -1.34$, $p = 0.19$). Preference for the NH ear is only present in the contralateral cortex where expected (CI-NH contrast estimate: ACipsi-CI: -2.87, $p=0.047$; ACipsi-NH: -0.32, $p=0.82$).
